# Temporal consistency of neurovascular components on awakening: preliminary evidence from electroencephalography, cerebrovascular reactivity, and functional magnetic resonance imaging

**DOI:** 10.3389/fpsyt.2023.1058721

**Published:** 2023-05-05

**Authors:** Ai-Ling Hsu, Ming-Kang Li, Yi-Chia Kung, Zhitong John Wang, Hsin-Chien Lee, Chia-Wei Li, Chi-Wen Cristina Huang, Changwei W. Wu

**Affiliations:** ^1^Bachelor Program in Artificial Intelligence, Chang Gung University, Taoyuan, Taiwan; ^2^Department of Psychiatry, Chang Gung Memorial Hospital at Linkou, Taoyuan, Taiwan; ^3^Department of Radiology, Tri-Service General Hospital, Taipei, Taiwan; ^4^Graduate Institute of Mind, Brain and Consciousness, Taipei Medical University, Taipei, Taiwan; ^5^Department of Psychiatry, School of Medicine, College of Medicine, Taipei Medical University, Taipei, Taiwan; ^6^Research Center of Sleep Medicine, Taipei Medical University Hospital, Taipei, Taiwan; ^7^Department of Radiology, Wan Fang Hospital, Taipei Medical University, Taipei, Taiwan; ^8^Brain and Consciousness Research Center, Taipei Medical University-Shuang Ho Hospital, New Taipei, Taiwan

**Keywords:** sleep, sleep inertia, simultaneous EEG-fMRI, psychomotor vigilance task, cerebrovascular reactivity, neurovascular coupling, EEG power

## Abstract

Sleep inertia (SI) is a time period during the transition from sleep to wakefulness wherein individuals perceive low vigilance with cognitive impairments; SI is generally identified by longer reaction times (RTs) in attention tasks immediately after awakening followed by a gradual RT reduction along with waking time. The sluggish recovery of vigilance in SI involves a dynamic process of brain functions, as evidenced in recent functional magnetic resonance imaging (fMRI) studies in within-network and between-network connectivity. However, these fMRI findings were generally based on the presumption of unchanged neurovascular coupling (NVC) before and after sleep, which remains an uncertain factor to be investigated. Therefore, we recruited 12 young participants to perform a psychomotor vigilance task (PVT) and a breath-hold task of cerebrovascular reactivity (CVR) before sleep and thrice after awakening (A1, A2, and A3, with 20 min intervals in between) using simultaneous electroencephalography (EEG)-fMRI recordings. If the NVC were to hold in SI, we hypothesized that time-varying consistencies could be found between the fMRI response and EEG beta power, but not in neuron-irrelevant CVR. Results showed that the reduced accuracy and increased RT in the PVT upon awakening was consistent with the temporal patterns of the PVT-induced fMRI responses (thalamus, insula, and primary motor cortex) and the EEG beta power (Pz and CP1). The neuron-irrelevant CVR did not show the same time-varying pattern among the brain regions associated with PVT. Our findings imply that the temporal dynamics of fMRI indices upon awakening are dominated by neural activities. This is the first study to explore the temporal consistencies of neurovascular components on awakening, and the discovery provides a neurophysiological basis for further neuroimaging studies regarding SI.

## Introduction

Every morning, upon opening our eyes in bed, we experience a certain period of time perceiving hypovigilance, cognitive impairments, and disoriented behavior; this phenomenon is termed as “sleep inertia” (SI). The difficulty of returning to vigilance may occur every day because the transition from sleep to wakefulness is never an on/off quick switch but is rather a sluggish dynamic process. A typical demonstration of hypovigilance in SI is through repeated observations of the slow, as compared to their presleep conditions, reaction time (RT) soon after awakening [usually using a psychomotor vigilance task (PVT) or auditory RT task] (1, 2). In general, the postsleep cognitive deficit recovers gradually as time passes until we feel rejuvenated with full control following a long sleep. SI can last roughly a few to thirty minutes (2–4), and such short-term hypovigilance period can be further lengthened among the participants with prior sleep deprivation, patients with obstructive sleep apnea, or those with narcolepsy (5, 6). In other words, the SI period involves a time-varying transition that alters an individual’s mental status from a sleeping unconscious state to an alert conscious state, depending on their neurophysiological conditions. The underlying brain-rebooting procedure of regaining cognitive performances, or even the consciousness, has attracted a series of neuroscience studies on SI. Previous studies have shown that immediately upon awakening, the sleep-like electroencephalography (EEG) features are prominent in SI, such as the persistent theta power (associated with deep sleep) and decreased beta (*β*) power (associated with wakefulness) (7, 8). EEG analyses have also presented an anterior-to-posterior spatial mismatch with prominent delta/theta power in the parieto-occipital lobe, indicating the carryover effect of sleep neurophysiology during SI. Nevertheless, the EEG studies are limited to delineating the spatial information of brain reorganizations in the post-awakening period.

For spatially localizing brain functionality in SI, Balkin et al., using H_2_^15^O positron emission tomography, studied the rapid recovery of cerebral blood flow (CBF) in the thalamus and a gradual CBF recovery in the anterior cortical regions (9), indicating post-awakening hemodynamic re-establishment. Based on similar hemodynamic perspectives, recent studies using functional magnetic resonance imaging (fMRI), in conjunction with EEG or polysomnography, have presented asynchronous brain-network reorganizations during SI. Our group first demonstrated that the functional connectivity pattern of the sensorimotor network (SMN) in the SI period after nocturnal sleep seemed disconnected, as if it remained disrupted in non-rapid eye movement (NREM) sleep, whereas the default-mode network (DMN) showed an intact connectivity pattern with quick recovery (10, 11). Probing the nap inertia after partial sleep deprivation, Vallat et al. demonstrated a distinction in between-network recovery between the participants awakened from deep sleep (NREM sleep stage 3, N3) and those awakened from light sleep (NREM sleep stage 2, N2) (12). On the basis of simultaneous EEG-fMRI recordings, Chen et al. showed that the strong correlation between the EEG vigilance index and fMRI frontoparietal network activity before sleep disappeared within the 5 min SI period after a 2 h nocturnal sleep (13). These neuroimaging studies indeed paved the way for new strategies to probe brain functional reorganization upon awakening from various neurophysiological angles (CBF, within-network connectivity, between-network connectivity, and EEG-fMRI associations).

After reading the slow recovery of brain functionality in the SI period, we found two obstacles impeded linking of these novel SI neuroimaging findings to the transient cognitive impairments. First, all the fMRI findings were based on the functional hyperemia or blood oxygenation level dependent (BOLD) principle (14, 15), which is an indirect measure of neural activities, and the spontaneous activities in the resting-state fMRI are also observed under the same BOLD assumptions. Considering the fMRI findings as the evidence of underlying neural activity can only be trusted when confirmed through neurovascular coupling (NVC) (16). However, our previous investigations revealed inconsistent dynamic changes between EEG (surrogate of neuron-related local field potential, LFP) and fMRI (neuron-related hemodynamic outcome based on BOLD principle) across NREM sleep stages, implying the neurovascular coupling during sleep may not be as static as that during the wakefulness (17). Thus, whether the assumption of static NVC holds in SI remains an open question. Second, the neuroimaging findings to date regarding SI have all been based on the brain connectivity in a “resting state” instead of involving cognitive engagements, which means that these findings cannot intuitively reflect cognitive performances in the SI period. Therefore, we aimed to solve these two difficulties regarding further SI investigations on brain reorganizations in this study.

**Figure 1:**
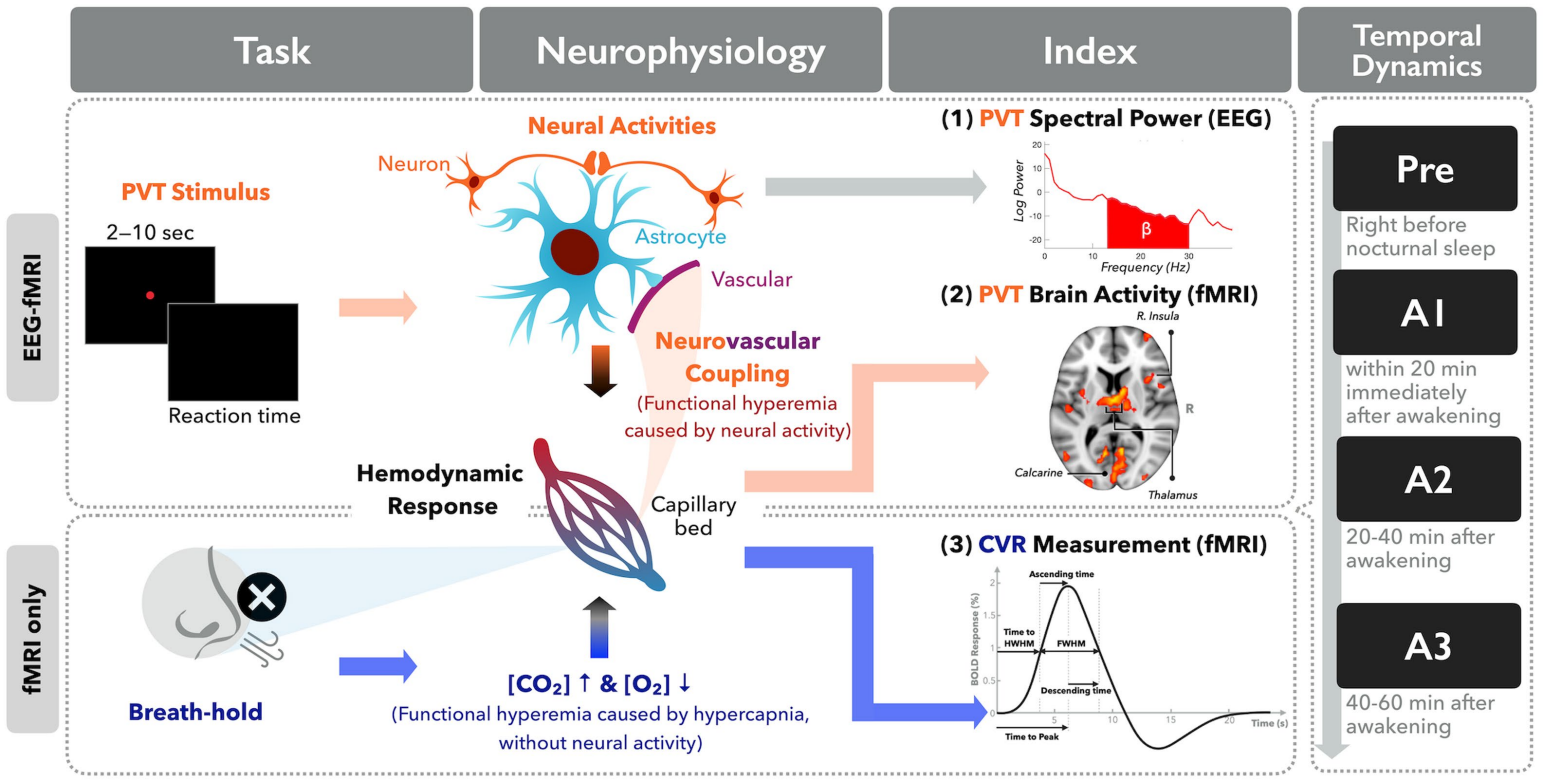
Diagram illustration of the neurophysiological concept underlying neuroimaging indices in this study.

Theoretically, NVC was affected by many microscopic factors such as adenosine, nitric oxide, lactate, etc. (18, 19); however, the quantification of these microscopic factors in the human brain is an arduous task. To evaluate the NVC assumption in SI from the neuroimaging perspective, we turned to evaluate the macroscopic brain signals from multi-modal neuroimaging methods through independent measures of EEG and BOLD-fMRI response. Meanwhile, we attempted to use the breath-hold task for inducing hypercapnia, termed as cerebrovascular reactivity (CVR, surrogate of pure hemodynamic response irrelevant to neural activity), immediately upon awakening from sleep (20, 21). Here the breath-hold CVR is regarded as an approach to probe the hemodynamic response function (HRF) irrelevant to the neural activities along the SI period. We hypothesize that the CVR pattern would remain the same across multiple measures between presleep and SI periods, indicating an unchanged NVC. Regarding cognitive associations, we had participants perform a modified PVT in order to measure vigilance in the SI period after nocturnal sleep, leveraging the technical advances of simultaneous EEG-fMRI recordings. Figure 1 delineates the concept of neurophysiology and experimental design in this study.

## Materials and methods

### Study participants

Fifteen adults aged between 20 and 40 years participated in this study and maintained consistent sleep–wake patterns for at least 3 days preceding the MRI scan. Their wake-sleep rhythms were monitored through wrist actigraphy (SOMNOwatchTM plus, SOMNOmedics GmbH, Randersacker, Germany). All participants self-reported to have ability to undergo EEG-fMRI scanning without history of neurological, or psychiatric diseases. Neither alcohol nor caffeinated products were allowed on the scanning day. The Pittsburgh Sleep Quality Index (PSQI) was administered to all participants to assess their sleep quality and disturbances over a 1-month period. All study procedures were approved by the Research Ethics Committee of National Taiwan University (Approval No. 201512ES054). Informed consent was obtained from all participants included in the study.

### Experimental design

The participants were instructed to lie supine in the MRI scanner. Following a 5 min anatomical scan, participants were required to perform four 20 min experimental sessions during SI. Considering that the duration of SI has been reported to be 15 to 30 min upon awakening (4, 5), the four sessions were designed to include one presleep (Pre) session as the baseline, and three post-awake (A1, A2, and A3) sessions. Specifically, the presleep session was set to 20 min before the averaged bedtime over the past week, and the first post-awake session was conducted after the maximum duration of 180 min of sleep. Each 20 min experimental session consisted of a 5 min resting state (RS), 6 min PVT, and 4 min CVR scan. The RS fMRI data were designed for other purposes beyond the scope of this work; thus, they are not reported here.

We designed a PVT task with a total of 72 trials in the current study to assess alertness during SI (22, 23). Participants were asked to passively view the visual stimuli and respond with a mouse click as soon as they saw a target. Each target was presented as a red solid circle with a 1 s duration, followed by a white cross fixation with a random time interval of 1 s to 7 s. Regarding the CVR scan at the end of each session, participants were asked to perform breath-hold (BH) tasks based on the instruction presented by visual stimuli to assess their CVR (24). The BH paradigm comprises an initial 6 s natural breathing period, followed by three blocks of alternations between 15 s BH and 45 s natural breathing, ending with a block of 15 s BH and 39 s natural breathing (25). A respiration monitoring device was utilized to confirm the participant’s compliance during the CVR scan. Cushions were provided to minimize head motion.

### Simultaneous EEG-fMRI recording

The simultaneous EEG-fMRI recordings were conducted for the four 20 min sessions. According to the international 10–20 system, the EEG data were recorded by a 32-channel MRI-compatible system, including two electrooculography (EOG) channels, two electromyography (EMG) channels, and one electrocardiogram (ECG) channel (Brain Products GmbH, Gilching, Germany). For the details setting of EEG recording, please refer to a previous study by Tsai et al. (11). Briefly, the impedances of the reference (FCz) and ground (AFz) channels were kept below 5 kΩ, yet those of other channels were kept below 15 kΩ. The EEG signals were synchronized with the MR trigger and recorded using BrainVision Recorder software (Brain Products) with settings of 5k Hz sampling rate and 0.1 μV voltage resolution. In addition, online filtering was applied with an analog band-pass filter (0.0159–250 Hz) and a 60 Hz notch filter. In addition, we carried out a three-end synchronization among MRI, EEG recording and task stimulation computers using the Brain Products Trigger Box and the software of E-Prime Extensions for Brain Products. The MR images were acquired by a 3T Tim Trio scanner (Siemens, Erlangen, Germany) with a 12-channel head coil, including a high-resolution T_1_-weighted anatomical images obtained using a 3D-MPRAGE sequence (TR/TE/TI = 1900 msec/2.28 msec/900 msec; flip angle = 9°; 176 slices with voxel size of 1 × 1 × 1 mm^3^), and functional scans using a T_2_^*^-weighted gradient-echo echo-planar imaging sequence (TR/TE = 2000 msec/30 msec; flip angle = 77°; 32 slices with 4 mm thickness and no gap; in-plane resolution = 3.44× 3.44 mm^2^). A total of 150, 180, 120 volumes were acquired for RS, PVT, and CVR scans, respectively.

### EEG analysis and sleep staging

Recorded EEG data was preprocessed offline using Brain Vision Analyzer 2.1 (Brain Products) and EEGLAB v13.6.5b (26). Analyzer with the average artifact subtraction method was used to remove artifacts induced by MR gradient and ballistocardiogram. In gradient-induced artifact removal, EEG data was up-sampled to 50k Hz. EEG data was then down-sample to 250 Hz to remove ballistocardiogram artifact. Subsequently, EEGLAB was used for the further four preprocess steps, including (1) bandpass filtering the EEG frequencies between 0.1 and 50 Hz, (2) rejecting noisy epochs (single TR per epoch) with the criterion 5 times of standard deviation above the mean of each channel (27), (3) re-referencing each EEG channel to the average over all EEG channels, and (4) utilizing temporal independent component analysis (ICA) to eliminate ICs with the characteristics similar to ECG, EMG, EOG channels. For the sleep staging, technicians scored EEG preprocessed data based on the American Academy of Sleep Medicine criteria (28), which used six EEG channels (F3, F4, C3, C4, O1, O2), two EOG channels, and two EMG channels for every time window with 30 s. After preprocessing, EEG data were epoched 250 milliseconds prior to PVT stimuli onset and 1750 milliseconds after. Power spectral density (PSD) was calculated using the psd_multitaper function in MNE-python (29), and then averaged across all epochs for each participant. The averaged PSD was further decomposed into four canonical frequency bands (delta: 0.5–4.5 Hz, theta: 4.5–7.5 Hz, alpha: 7.5–11.5 Hz, beta: 11.5–30 Hz) to assess the relative power across sessions.

### fMRI analysis and behavior indices

The PVT fMRI data were preprocessed using an in-house script based on AFNI (version number: 18.0.25) (30) through motion correction, slice-timing, alignment to the T_1_-weighted anatomical image, spatial smoothing with a 6 mm full width at half maximum (FWHM) Gaussian kernel, and spatial normalization into the standard Montreal Neurologic Institute (MNI) space. The first-level analysis was performed using the general linear model with 9 regressors, including one for onset timing of the corresponding 1 s stimuli convolved with the canonical hemodynamic response function, six for motion parameters, the other two for baseline intensity and linear trend. Next, only beta estimates in the presleep session were analyzed with second-level analysis using *3dttest++* to identify the brain regions that were significantly activated across participants. Regarding correction for multiple comparisons, the significant activations were corrected using the AFNI *3dClustSim* method with autocorrelation function (corrected *p* < 0.05), wherein the parameter setting was a combination of an uncorrected threshold of *p* < 0.0005 and an individual cluster size of 46 contiguous voxels. Among significant clusters activated in the presleep session, the three regions of interest (ROIs) that most related to the PVT task were further selected for the ROI analysis. Along with PVT experiments, behavioral indices were measured in terms of accuracy and mean response time (RT). Only hit trials, in which participants responded within 0.95 s, were used to calculate the latter two indices.

The CVR data were preprocessed using IClinfMRI (31) with the default setting and normalized into the MNI space using SPM12 (6685) (The Wellcome Centre for Human Neuroimaging, UCL, London, United Kingdom). Magnitudes of each preprocessed data were then normalized to their voxel-wise baseline signal to yield percent signal change. To estimate the temporal characteristics of CVR upon awakening, the CVR response function was averaged across four blocks from the BH onset (0 s) to the 54th seconds within ROIs selected based on presleep PVT activations. Subsequently, each averaged CVR response function was nonlinearly fitted with a canonical dual-gamma function using Matlab’s *lsqcurvefit* function (The MathWorks, Inc., Natick, MA, United States). Accordingly, the fitted curves were used to estimate temporal characteristics, including time-to-half width half maximum of the peak, ascending time, time to peak, FWHM of the peak, and descending time (see Supplementary Figure S1).

### Statistical analysis

A nonparametric repeated-measure Friedman test and false discovery rate (FDR)-corrected *post hoc* test were performed using Python (SciPy version 1.9.1) to determine significant differences across four sessions in behavior, EEG, PVT, and CVR indices. The significance level was set as *p* < 0.05.

## Results

A total of 15 participants aged between 20 and 31 years (eight females; mean age = 24.9 ± 4.0 years; all obtaining at least a high school degree) completed the EEG-fMRI recording, and three participants had incomplete PVT data. Accordingly, all participants were used in the neuron-irrelevant CVR analysis, but only 12 participants were used for the PVT-induced fMRI and behavior analysis. Supplementary Table S1 shows the demographic and sleep characteristics of each participant. All participants reported at least 20% sleepiness, with their average being 64.3 ± 19.53%. The average PSQI was 4.40 ± 2.77, and total sleep time was 24.97 ± 43.96 min. However, three participants out of twelve, who self-reported being able to sleep inside the scanner with zero total sleep time according to sleep scoring, were further excluded in the EEG analysis.

Compared to an accuracy of 0.97 ± 0.08 in the PVT task before sleep, nonsignificant reduced postsleep accuracies of 0.95 ± 0.16, 0.94 ± 0.14, and 0.95 ± 0.11 were found for A1, A2, and A3, respectively (Supplementary Figure S2). By contrast, nonsignificant increased RTs in the postsleep sessions (358 ± 66, 363 ± 70, and 356 ± 57 milliseconds for A1, A2, and A3, respectively) were observed; for comparison, the presleep RT was 338 ± 49 milliseconds.

**Figure 2:**
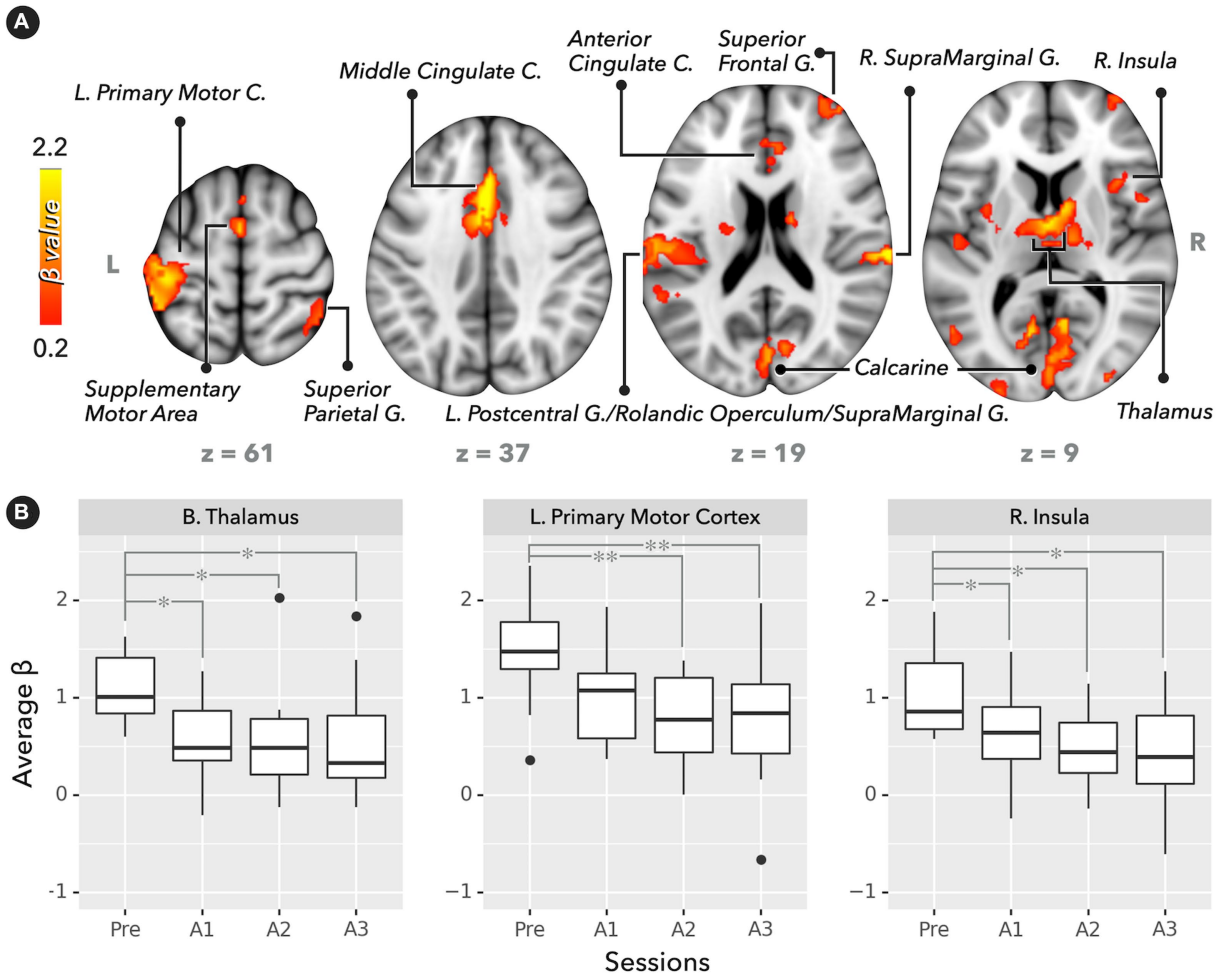
Brain activation map and regional BOLD response (average *β* values) in PVT (*n* = 12). **(A)** Significant activation map in the presleep session (corrected *p* < 0.05). **(B)** BOLD responses for bilateral thalamus, left primary motor cortex, and right insula. Compared with the presleep session, BOLD responses significantly decreased after sleep (FDR-corrected *post hoc* ^*^p < 0.05 and ^**^p < 0.01).

**Figure 3:**
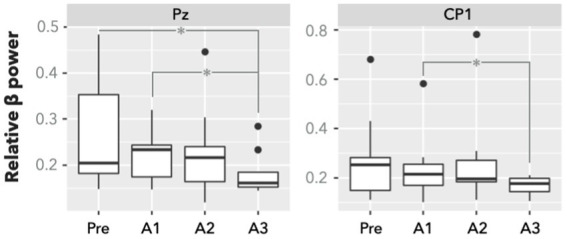
Relative *β* power of EEG electrodes of Pz and CP1 in PVT (*n* = 9). There is a significant difference between presleep and the third 20-min sessions after sleep (A3) in Pz. Compared with the first session after sleep (A1), the power at the A3 session significantly decreased in both Pz and CP1. Significance was determined by FDR-corrected *post hoc* ^*^p < 0.05.

Figure 2A depicts the significant recruitment of brain regions engaged in PVT tasks during the presleep session (corrected *p* < 0.05). The brain regions included the supplementary motor area, anterior/middle cingulate cortex, left primary motor cortex, right superior frontal gyrus, right insula, right superior parietal gyrus, bilateral supramarginal, gyrus and bilateral thalamus. For the ROI analysis, Figure 2B demonstrates that the regional BOLD response (average *β* values) significantly decreased in the postsleep sessions (FDR-corrected *post hoc* ^*^p < 0.05 and ^**^p < 0.01) compared to the presleep session for the bilateral thalamus, left primary motor cortex, and right insula. In addition to the PVT-induced fMRI results, a significant difference was evident in the relative *β* power measured by the EEG at electrodes of Pz and CP1 (Figure 3). The group means of relative *β* power in Pz were 0.26 ± 0.12, 0.23 ± 0.06, 0.23 ± 0.10, and 0.18 ± 0.05 for the Pre, A1, A2, and A3 sessions, respectively; those in CP1 were 0.28 ± 0.18, 0.24 ± 0.14, 0.27 ± 0.20, and 0.17 ± 0.04 for the four sessions, respectively. Specifically, in comparing with the presleep session at Pz, the relative *β* power in A3 was significantly decreased. At both Pz and CP1, the relative *β* power in A3 was significantly smaller than that in A1.

Regarding the neuron-irrelevant CVR, Table 1 lists the CVR temporal indices across sessions for the three selected ROIs. The Friedman test found that the FWHM within the bilateral thalamus was significant across sessions. The *post hoc* test with FDR correction showed that the FWHM was significantly narrower in A3 than in the presleep session (*p =* 0.03). However, there were nonsignificant *post hoc* differences in the CVR indices within both the left primary motor cortex and right insula across sessions.

**Table 1:** Temporal characteristics of breath-hold CVR estimated from fitted HRF within region of interests.

	Pre	A1	A2	A3	Friedman test
*Left primary motor cortex*					
Time to HWHM	29.39 ± 6.27	26.31 ± 1.94	25.71 ± 1.86	25.83 ± 1.84	*χ*^2^ = 3.96, *p =* 0.27
Ascending time	3.46 ± 1.46	4.89 ± 2.14	6.15 ± 6.00	4.45 ± 1.94	*χ*^2^ = 9.00, *p =* 0.03^*^
Time to peak	32.84 ± 5.50	31.19 ± 3.27	31.86 ± 5.72	30.28 ± 1.98	*χ*^2^ = 0.04, *p =* 1.00
Descending time	4.82 ± 2.96	6.31 ± 4.50	7.11 ± 6.07	4.97 ± 1.92	*χ*^2^ = 1.64, *p =* 0.65
FWHM	8.28 ± 3.97	11.2 ± 5.52	13.26 ± 7.75	9.42 ± 2.51	*χ*^2^ = 5.64, *p =* 0.13
*Bilateral thalamus*					
Time to HWHM	25.42 ± 1.83	25.77 ± 1.81	25.83 ± 1.37	26.05 ± 1.51	*χ*^2^ = 2.60, *p* = 0.46
Ascending time	5.09 ± 1.27	4.69 ± 1.76	4.34 ± 0.85	4.17 ± 1.36	*χ*^2^ = 8.68, *p* = 0.03^*^
Time to peak	30.51 ± 1.99	30.46 ± 2.00	30.17 ± 1.91	30.22 ± 1.95	*χ*^2^ = 0.26, *p* = 0.97
Descending time	6.53 ± 1.99	6.03 ± 3.12	6.86 ± 5.03	4.85 ± 1.80	*χ*^2^ = 7.51, *p* = 0.06
FWHM	11.62 ± 2.58	10.72 ± 3.94	11.21 ± 5.74	9.02 ± 2.72^*^	*χ*^2^ = 9.00, *p* = 0.03^*^
*Right insula*					
Time to HWHM	22.3 ± 1.47	23.05 ± 1.23	22.47 ± 2.26	23.31 ± 1.67	*χ*^2^ = 2.25, *p* = 0.52
Ascending time	6.38 ± 2.04	5.32 ± 1.38	5.51 ± 2.56	4.9 ± 1.10	*χ*^2^ = 3.56, *p* = 0.31
Time to peak	28.68 ± 1.75	28.37 ± 1.14	27.97 ± 1.4	28.21 ± 1.45	*χ*^2^ = 8.84, *p* = 0.03^*^
Descending time	8.09 ± 2.05	6.98 ± 1.78	8.1 ± 3.89	6.85 ± 2.72	*χ*^2^ = 5.48, *p* = 0.14
FWHM	14.47 ± 3.64	12.3 ± 2.39	13.61 ± 6.34	11.75 ± 3.37	*χ*^2^ = 5.56, *p* = 0.14

Time unit is second. Data is summarized as mean ± standard deviation. Pre, presleep session; A1/A2/A3, the first/second/third 20-min session after sleep; FWHM, full width half maximum of the peak; HWHM, half width half maximum of the peak. The statistical significance between Pre and A3 of *p* < 0.05 with FDR *post hoc* tests is marked as ^*^.

## Discussion

This is the first attempt using the EEG-fMRI fusion technique to study the neuro-vascular variations during sleep inertia. Because of the difficulty of NVC quantification, we bypassed the complex neurophysiological mechanisms within NVC and targeted on observing the time-varying consistency of separate NVC components during the sleep inertia period. The separate NVC components include EEG signals (neuron-related local field potential, LFP), CVR (neuron-irrelevant hemodynamic response function) and BOLD response (neuron-related HRF) along the awakening time. We hypothesize that if the NVC stays intact during sleep inertia, EEG *β* power and BOLD response would show a consistency among temporal dynamics, but the neuron-irrelevant CVR would remain time-invariant across different time points on awakening. Our results confirmed this hypothesis because both EEG *β* power and BOLD response showed significant reductions upon awakening, as compared with the presleep condition. For the CVR, we fitted multiple temporal characteristics to the hemodynamic responses irrelevant to the neural activity under the BH task and found that the induced HRF did not show prominent changes before and after sleep, even though the baseline CBF varied during the SI period (9). In fact, although the thalamus exhibited a shorter FWHM upon awakening in the *post hoc* test, the overall CVR temporal patterns remained untouched without presleep/postsleep differences in the BOLD fMRI response and EEG *β* power. Thereby, the temporal patterns of EEG, CVR and fMRI preliminarily indicated that the BOLD responses upon awakening were contributed from neural activities, implying that the static NVC presumption still holds upon awakening after sleep.

In lack of task engagements, prior SI-based neuroimaging studies could not approach NVC on awakening. Here we conducted PVT experiments in the SI period using simultaneous EEG-fMRI recordings, which was carried out through a three-end synchronization among MRI, EEG recording and task stimulation computers. This is the first attempt to conduct the task-based fMRI upon awakening, linking cognitive performance with the fMRI brain mapping in the SI period. Furthermore, we measured multiple neuroimaging indices (EEG, CVR, and fMRI) to approach NVC through observing their temporal consistency (as shown in Figure 1). Unlike calibrated methods using mathematical modeling (18), this act of observing temporal consistency is imperative in this study because during SI, both neural activity and BOLD response were all varying along with time without a fixed reference for calibration (12, 13). Thus, we bypassed the neurophysiological modeling and turned to measure the macroscopic neuroimaging indices with task engagements repeatedly for estimating the both ends of neurovascular components, according to previous literature (17, 32). However, using this strategy we only provided preliminary evidence of static NVC on awakening with limited sample size. Further investigations with microscopic NVC-related factors are warranted to validate the static NVC assumption in the period of sleep inertia.

### Neurovascular coupling before sleep and after awakening

Previous fMRI investigations have all been based on the presumption of NVC and the BOLD principle (33). However, recent studies have suggested that the presumption might not be as valid as our prior expectation, even in the normal human participants without neuropathology. For example, Czisch et al. observed different patterns of fMRI activity between wakefulness (positive activity) and sleep stages (negative response) in response to the same acoustic stimuli (task) (34), and our recent studies demonstrated the temporal inconsistency between EEG and fMRI indices (power and connectivity) across sleep-wake conditions (resting) (17). Both studies highlighted the possibility of an altered neurovascular relationship in nocturnal sleep. The neurovascular relationship on awakening has not yet been explored. To understand how the brain recovers its functionality and consciousness after sleep, a critical next step is to determine whether the NVC assumption holds true during the SI period. To examine NVC through the resting-state analysis is not an easy task due to the lack of prominent targets in terms of both neural activity and the followed BOLD responses. Therefore, we utilized the PVT to probe vigilance upon awakening, which is a commonly adopted approach in the SI literature. At the current stage, the neuroimaging field lacks a quantitative index for NVC; thus, we adopted a qualitative examination method for NVC, observing the between-session consistency in the combined neurovascular responses (fMRI BOLD responses), vascular components (CVR temporal characteristics), and neural components (EEG *β* power). For the vascular component, we regarded the BH-induced CVR time curves as a surrogate for BOLD response without neural activity, and we carefully checked the CVR temporal characteristics through data fitting. The results indicated that the thalamus might be the only brain region with prominent changes in CVR temporal characteristics, especially for FWHM in thalamus. However, the cross-sessional patterns of FWHM were unlike those of the EEG *β* power or fMRI brain activity; the postsleep variation in FWHM was not prominent in other brain regions. Therefore, we could approximately conclude that the CVR function did not vary before and after sleep during the SI period. For the neural component of PVT, we found that the relative *β* power presented differently between the presleep session and A3 (Pz), as well as between A1 and A3 (Pz and CP1). As the EEG *β* power is generally regarded as an index of vigilance (8, 12, 35), we can infer that immediately after awakening, the nonsignificantly enhanced *β* power reflected the slight elevation in vigilance of the participants, but it decreased along the waking time, reaching a trough at A3. By contrast, upon awakening, the relative delta power increased with marginal significance in O2 (*χ*^2^ = 9.1, with *post hoc* FDR-corrected *p* = 0.078 for Pre–A3, A1–A2 and A2–A3), and FC5 (*χ*^2^ = 8.3, with *post hoc* tests of *p* > 0.195, FDR-corrected), indicating the extended sleepiness even after awakening. The findings of presleep/postsleep differences resembled the cross-session results of fMRI BOLD responses, which supports the idea of static NVC upon awakening.

### Sleep inertia under insufficient sleep

The multiple repeated-measure design after awakening is a common setting in previous SI-based studies (7, 9, 12). We originally expected that the RTs and brain activities would gradually recover close to the presleep condition along with time; however, our findings only identified the difference between presleep and postsleep, without differences between A1 and A3, for all indices of RT, fMRI activity, and EEG power. The unusual cross-session pattern after awakening might be an indication of extended sleepiness in the 1 h awakening period from a maximum 3 h sleep inside the MRI scanner (reflected by low *β* power and high delta power). One reason for this outcome may be the irregular waking time (approximate 3–4 a.m.) relative to the participants’ normal sleep-wake rhythm; the induced partial sleep deprivation might have led to an enhancement of the SI severity (5). The constrained body position inside the MRI scanner might be another influential factor for participants, as they cannot move during the 1 h scan after awakening, which may have induced sleepiness after a certain period of time. Third, the current results included the average EEG/fMRI/RT scores in sleep (*n* = 12), yet three of them did not show objective sleep signatures in sleep scoring. If we present the datasets only with objective sleep scoring (*n* = 9, see Supplementary Figure S3), the cross-session SI pattern is slightly evident in the fMRI effect size, which confirms the necessity of collecting the datasets with objective sleep scorings for SI investigations.

### Limitation

The major limitation of this study was the insufficient sample size to apply the parametric statistical tests. Originally, we collected 32 participants in the protocol; however, due to certain technical obstacles (e.g., loss of synchronization markers, unable to record PVT-RTs, and disruptive motion in sleep), the number of participants completing the CVR with simultaneous EEG-fMRI recordings was only 15, and that completing both the PVT and CVR was 12. After excluding those who did not present objective sleep signatures, the number decreased to nine. Due to the limited sample size, we only provided a preliminary glimpse on the NVC upon awakening for future neuroimaging investigations, and we could not classify the data into groups of different sleep stages that participants were awakened from, which is a necessary step for future studies. Finally, neurons at different brain locations may recover at different speeds in SI (11), or with different NVC (36). However, we only focused on the PVT-related brain regions (thalamus, insula, and motor cortex) in this study based on the task engagements. Future studies are warranted to continue surveying functional reorganization at different brain regions immediately after sleep.

## Conclusion

Sleep inertia is the period that involves cognitive impairments immediately after awakening from sleep. Previous papers have discussed hypovigilance in task engagement, but recent neuroimaging studies regarding SI have focused on the brain connectivity in the resting state. This mismatch as well as the presumption of static NVC before and after sleep are two major concerns in this field. Therefore, we firstly addressed brain activity in response to task performances within 1 h of awakening. Furthermore, we preliminarily probed the appropriateness of assuming the static NVC in presleep and postsleep conditions, which may facilitate the neuroimaging studies of SI in the future.

## Data availability statement

The original contributions presented in the study are included in the article/Supplementary material, further inquiries can be directed to the corresponding authors.

## Ethics statement

The studies involving human participants were reviewed and approved by the Research Ethics Committee of National Taiwan University (Approval No. 201512ES054). The patients/participants provided their written informed consent to participate in this study.

## Author contributions

C-WH, H-CL, and CW initiated the concept and experimental design. A-LH, M-KL, and CW wrote the manuscript. CW, Y-CK, and A-LH designed the study. M-KL, Y-CK, and C-WL collected the data. A-LH, M-KL, and ZW analyzed the data. All authors contributed to the article and approved the submitted version.

## Funding

This study was supported by the funding from the Taiwan National Science and Technology Council (105-2628-B-038-013-MY3, 108-2410-H-038-007, and 111-2222-E-182-001-MY3), Taiwan Ministry of Education (DP2-110-21121-01-N-06-01), and Taipei Medical University-Wanfang Hospital (110TMU-WFH-17). This manuscript was edited by Wallace Academic Editing.

## Conflict of interest

The authors declare that the research was conducted in the absence of any commercial or financial relationships that could be construed as a potential conflict of interest.

## Publisher’s note

All claims expressed in this article are solely those of the authors and do not necessarily represent those of their affiliated organizations, or those of the publisher, the editors and the reviewers. Any product that may be evaluated in this article, or claim that may be made by its manufacturer, is not guaranteed or endorsed by the publisher.

## Supplementary material

The Supplementary material for this article can be found online at: https://www.frontiersin.org/articles/10.3389/fpsyt.2023.1058721/full#supplementary-material
