# Supplementary material for: Temporal consistency of neurovascular components on awakening: preliminary evidence from electroencephalography, cerebrovascular reactivity, and functional magnetic resonance imaging
Source: Front Psychiatry. 2023 May 5;14:1058721. doi: 10.3389/fpsyt.2023.1058721 (PMC10196490; doi:10.3389/fpsyt.2023.1058721)
Supplement: Supplementary file 1 [file Data_Sheet_1.PDF]

Supplementary TABLE 1 Demographic and Sleep Characteristics

Subject no.	Sex	Age (yr)	Education years	PSQI	Self-reported Sleepiness (%)	Total Sleep Time (min)	Wake (min)	N1 (min)	N2 (min)	N3 (min)
1	F	26	12	0	90	161	14	98	30	33
[a]2	M	32	23	1	80	0	127	0	0	0
3	F	30	20	5	20	0	90	0	0	0
4	M	28	19	4	60	8.5	34	9	0	0
5	F	22	16	9	60	0	50	0	0	0
6	M	21	15	5	70	0	110	0	0	0
7	F	21	14	1	70	16	161	16	0	0
8	M	21	15	7	70	26	151	18	8	0
9	F	27	17	6	60	82.5	33	2	81	0
10	F	28	21	9	40	22.5	126	4	19	0
[a]11	F	22	17	5	85	21.5	147	21	1	0
12	F	31	17	3	90	14.5	94	15	0	0
13	M	21	16	3	70	2	76	2	0	0
14	M	21	16	2	60	20	157	5	16	0
[a]15	M	22	16	6	40	0	65	0	0	0

Note: [a]Participant had CVR but PVT data. PSQI = Pittsburgh Sleep Quality Index.

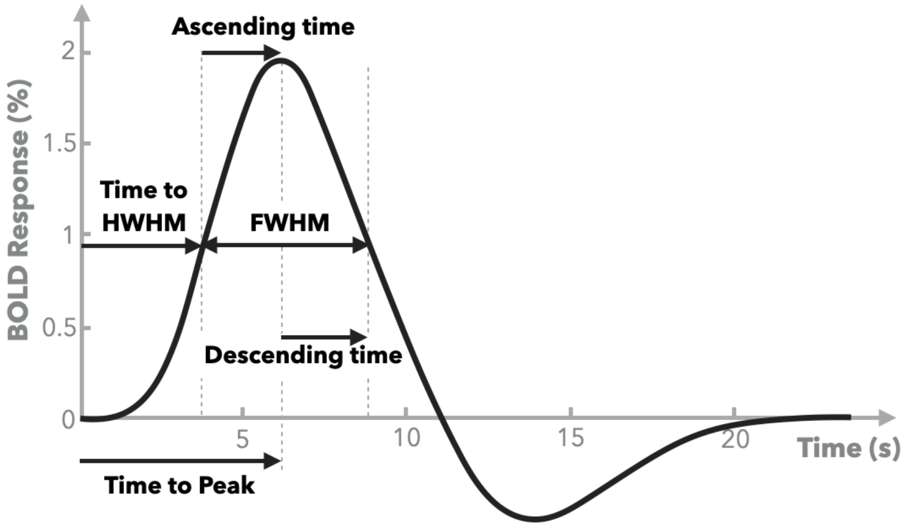

Supplementary Figure 1. *Illustration of Hemodynamic Response Function (HRF) modeled by canonical dual-gamma functions. Parameters specifying the HRF are illustrated.*

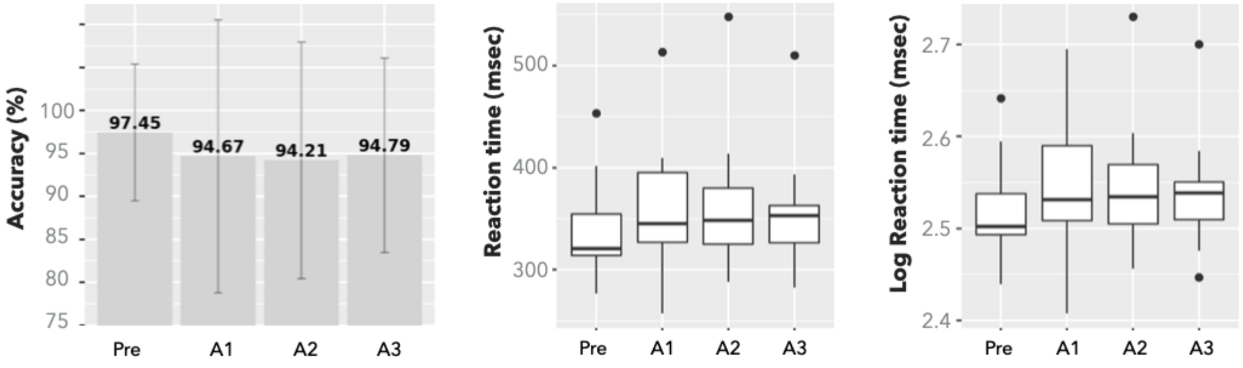

Supplementary Figure 2. *Non-significant behavior indices of accuracy, reaction time, and log reaction time across 4 sessions (n=12).*

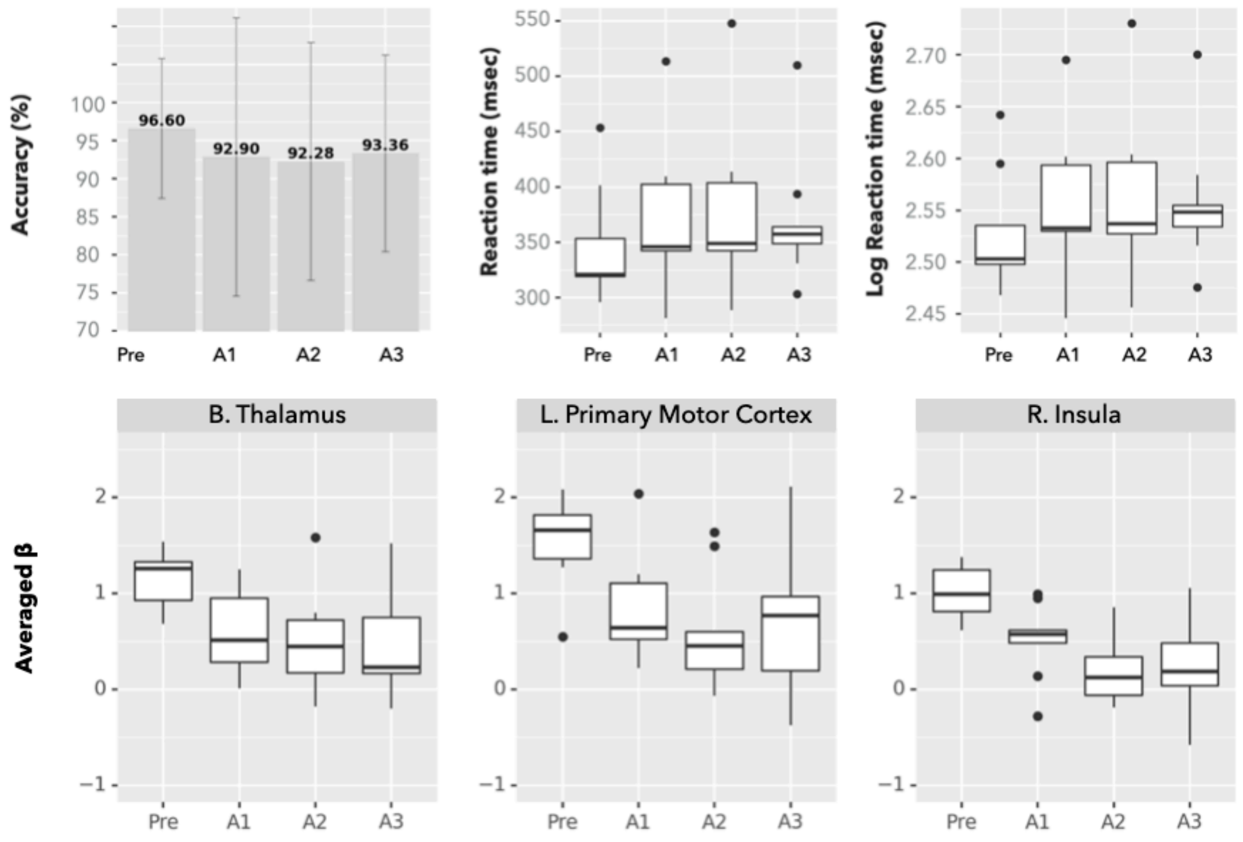

Supplementary Figure 3. *Non-significant behavior indices of accuracy, reaction time, and log reaction time as well as PVT-induced β values across 4 sessions (n=9).*
